# Approaches to reduce succinate accumulation by restoration of succinate dehydrogenase activity in cultured adrenal cells

**DOI:** 10.1242/jcs.263925

**Published:** 2025-05-12

**Authors:** Fatimah Al Khazal, Leili Rahimi, Fan Feng, Nicole A. Becker, Clifford D. Folmes, Judith Favier, L. James Maher

**Affiliations:** ^1^ Department of Biochemistry and Molecular Biology, Mayo Clinic College of Medicine and Science, Rochester, MN 55905, USA; ^2^ Department of Biochemistry and Molecular Biology, Department of Cardiovascular Medicine, Mayo Clinic College of Medicine and Science, Scottsdale, AZ 85259, USA; ^3^ Inserm, Centre de recherche des Cordeliers, Université Paris-Cité, Sorbonne Université, Equipe Labellisée Ligue contre le Cancer, 75006, Paris, France

**Keywords:** Paraganglioma, Pheochromocytoma, Succinate dehydrogenase, Riboflavin

## Abstract

The rare human neuroendocrine tumors pheochromocytoma and paraganglioma (PPGL) can result from loss of mitochondrial succinate dehydrogenase. The resulting succinate accumulation is tumorigenic in certain neuroendocrine cells. Here, we explore two theoretical approaches to mitigate tumorigenic succinate accumulation in a cell culture model of PPGL. We first study a gene replacement strategy using transposition technology, and conclude that many of the changes in mitochondrial morphology, oxidative cell metabolism and succinate accumulation can be reversed by this process. We then investigate whether riboflavin supplementation has the potential to rescue succinate dehydrogenase activity in the intact SDHA catalytic subunit to suppress succinate accumulation even in the absence of SDHB. We show that this latter strategy is not successful.

## Teaser

**Summary:** Tumors can be caused by loss of succinate dehydrogenase (SDH), resulting in oncogenic succinate accumulation. This imbalance can be reversed by restoring the missing SDH subunit, but not by riboflavin supplementation to boost SDH catalytic activity.

## INTRODUCTION

Carriers of loss-of-function alleles in genes encoding the four succinate dehydrogenase (SDH) subunits (A–D) exhibit a heightened risk for pheochromocytoma and paraganglioma (PPGL), rare neuroendocrine tumors of chromaffin cells (Gimenez-Roqueplo et al., 2023; Morin et al., 2020; Buffet et al., 2020; Pang et al., 2019; Her and Maher, 2015). Tumorigenesis begins with the somatic loss of the remaining functional gene copy, resulting in a cell deficient in SDH activity. SDH thus acts as a tumor suppressor in this context.

**Fig. 1.**
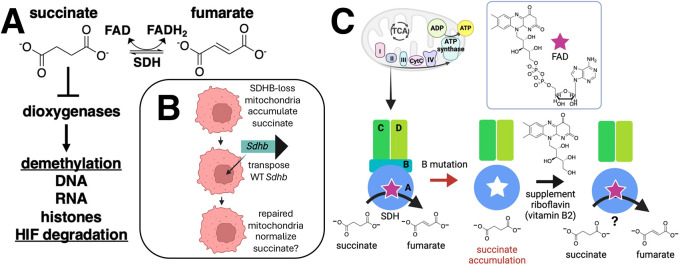
**Concepts tested in this work.** (A) Oxidation reaction catalyzed by SDH, yielding fumarate and FADH_2_. In the absence of SDH activity, accumulating succinate acts as a competitive inhibitor of dozens of dioxygenases that depend on O_2_, Fe^2+^ and 2-ketoglutarate to demethylate the indicated macromolecules as well as hydroxylate hypoxia-inducible factor (HIF) triggering its degradation. Inhibition of these dioxygenases is believed to be tumorigenic in certain neuroendocrine cells. (B) Hypothesis I: therapeutic restoration of WT *Sdhb* DNA. SDHB-loss cells will normalize succinate levels. Created in BioRender by Maher, J., 2025. https://BioRender.com/ibzyo5z. This figure was sublicensed under CC-BY 4.0 terms. (C) Hypothesis II. Loss of SDHB from otherwise normal mitochondria releases intact but poorly-flavinylated SDHA subunits whose activities can be rescued by riboflavin supplementation, moderating succinate levels in the absence of complex II function. Created in BioRender by Maher, J., 2025. https://BioRender.com/salj3ys. This figure was sublicensed under CC-BY 4.0 terms.

An interesting PPGL model is provided by immortalized mouse adrenal medulla-derived cell lines termed imCCs (Letouze et al., 2013). Compared to wild-type (WT) imCCs, the SDHB-loss imCCs are larger, grow more slowly, display swollen and deformed mitochondria, show >130-fold succinate accumulation and display an altered metabolism downregulating TCA cycle intermediates but preserving some complex I activity in the electron transport chain (Kluckova et al., 2020; Al Khazal et al., 2024). Among several mechanisms proposed to link SDH deficiency with PPGL tumorigenesis, the accumulation of succinate as an oncometabolite is a leading hypothesis (Selak et al., 2005) (Fig. 1A). Succinate is a competitive inhibitor of dozens of mammalian oxygen-, iron- and 2-ketoglutarate-dependent dioxygenases, and is responsible for a multitude of cellular functions including epigenetic demethylation of DNA, RNA and histones, as well as marking hypoxia-inducible transcription factor (HIF) subunits for degradation (Losman et al., 2020; Kaelin, 2009). Approaches to suppress succinate accumulation might therefore be attractive in early stages of PPGL prevention or management.

Here, we describe two attempts intended to therapeutically suppress succinate accumulation in SDHB-loss imCCs. First, we envisioned a gene therapy approach to replace defective SDHB with a rescuing WT *Sdhb* or negative control WT *Sdhc* transgene via transposon technology (Fig. 1B). We opted for *Sdhc* replacement as an uncomplicated negative control, although future studies could compare this result to replacement with validated loss-of-function missense *Sdhb* constructs. We document the success of this approach by demonstrating the reversal of many of the cellular pathologies associated with SDHB loss, most notably succinate accumulation. Second, riboflavin therapy has previously been suggested to enhance residual SDHA subunit flavinylation when other SDH subunits are missing (Fig. 1C), partially restoring succinate oxidation in SDH defective cells, and possibly reducing succinate levels by supporting conversion of accumulated succinate to fumarate even in the absence of SDHB (Maio et al., 2016). We tested this by treating WT and SDHB-loss imCCs with various concentrations of riboflavin. We report that this tactic did not suppress succinate accumulation in this model.

## RESULTS AND DISCUSSION

### Attempts to rescue SDH activity in cultured SDHB-loss imCCs by gene replacement

**Fig. 2.**
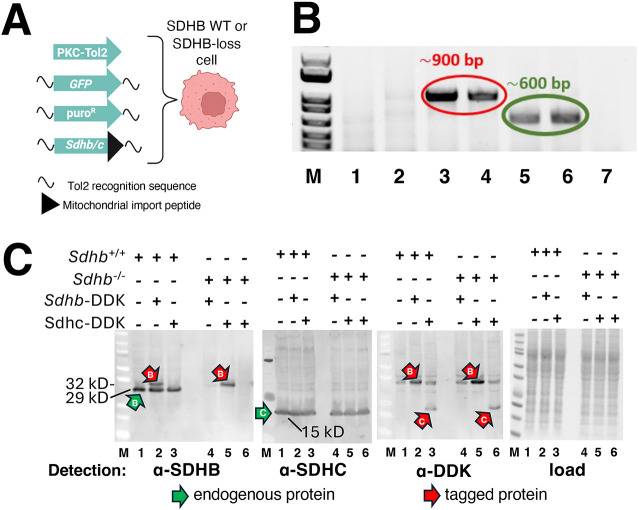
**Testing rescue of SDHB-loss cells by *Sdhb* gene replacement.** (A) Simultaneous transposition of the indicated four transgenes into SDHB WT or SDHB-loss imCCs. Tol2 recognition and mitochondrial import peptide sequences are noted. Created in BioRender by Maher, J., 2025. https://BioRender.com/ibzyo5z. This figure was sublicensed under CC-BY 4.0 terms. (B) PCR confirmation of successful transposition. Lanes 1,2, *Sdhb^−^*^/−^ imCCs without transposition of rescuing DNA. Lanes 3,4 show PCR confirmation of SDHB transgene transposition without (lane 3) or with (lane 4) GFP DNA. Corresponding data for SDHC rescue plasmids in lanes 5,6. Lane 7, no DNA added before PCR. (C) Western blotting with protein detection by the indicated antibodies or Imperial stain loading control (below) against SDHB, SDHC or MYC-DDK epitope tag indicating endogenous and epitope-tagged SDH subunit forms. Predicted molecular masses: WT SDHB unprocessed (32 kDa) and processed (29 kDa); epitope-tagged SDHB: unprocessed (35 kDa) and processed (32 kDa); WT SDHC unprocessed (18 kDa) and processed (15 kDa); epitope-tagged SDHC unprocessed 22 kDa) and processed (19 kDa). All detected SDH species correspond to processed forms. Images representative of three experimental repeats.

**Fig. 3.**
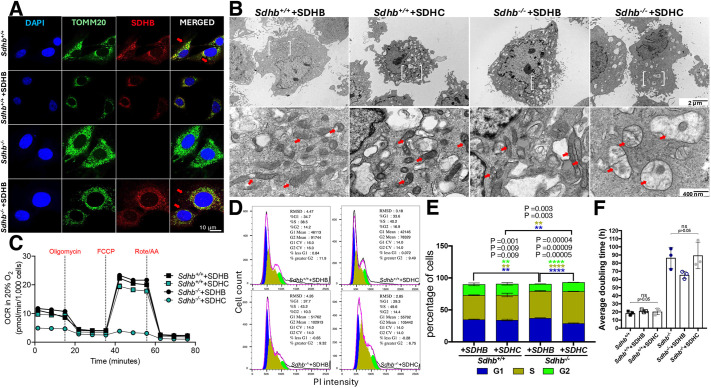
**Transposed SDHB rescues imCC phenotypes.** (A) Mitochondrial localization of transposed SDHB protein (red) based on colocalization with TOMM20 (green) as a mitochondrial marker. Red arrows compare location of extra-mitochondrial SDHB in rescued *Sdhb^−/−^* compared to WT imCCs. (B) *Sdhb* transposition substantially normalizes mitochondrial ultrastructure in SDHB-loss imCCs. White brackets indicate expanded areas in 400 nm micrographs (below) and red arrows indicate examples of mitochondria. Images in A and B representative of three experimental repeats. (C) Metabolic profiles of the indicated cell lines using a Seahorse mitochondrial stress test performed in 20% O_2_. Results of replicates and statistical significance is reported in Fig. S1. (D) Flowjo-generated cell cycle histograms showing representative data for the percentage cells in cell cycle phases (G1, S or G2) based on DNA content (PI intensity) using a Watson analysis model (*n*=20,000 cells per sample) for the indicated cell lines. RMSD values and statistical measures are reported for each graph and are representative across all three replicates for each line. (E) Mean percentage of cells in each of the indicated cell cycle phases (G1, S or G2) for each of the indicated lines plotted as mean±s.e.m. (*n*=3). DNA content not assigned to any of the indicated phases is not counted, bringing the total percentage below 100%. *P*-values were determined using Bonferroni correction for multiple *t*-tests and the number of asterisks indicates degree of significance. (F) Mean±s.e.m. cell doubling time for each indicated cell line. No statistically significant differences were noted upon *Sdhb* or *Sdhc* rescue.

We tested a gene therapy approach (Fig. 1B) to replace defective SDHB with a rescuing transgene. Initial lentiviral gene transduction attempts were not successful, as SDHB-loss imCCs were found to resist lentiviral transduction. SDHB-knockout (KO) imCCs therefore were transposed with DNA encoding *Sdhb* or *Sdhc* cDNAs via TOL2 transposon technology (Clark et al., 2011) (Fig. 2A), confirmed by PCR (Fig. 2B) and analyzed by western blotting to monitor protein processing and levels (Fig. 2C). Western blotting showed that both WT and SDHB-loss imCCs successfully expressed MYC-DDK epitope-tagged SDHB transgene products at a molecular mass consistent with processed removal of mitochondrial targeting peptides (Fig. 2C, anti-SDHB panel lanes 2 and 5, anti-DDK panel lanes 2 and 5). We note that a nonspecific anti-DDK tag-reactive protein is detected at a mass similar to tagged SDHB even in control extracts (Fig. 2C, anti-DDK panel lanes 1, 3, 4, 6) but signal from transposed tagged SDHB protein is evident by its greater intensity relative to this background signal. Whereas a monoclonal anti-SDHC antibody detected WT SDHC but not the C-terminal epitope-tagged form (Fig. 2C, anti-SDHC panel), probing with anti-DDK antibody demonstrated successful transposition and processing of epitope-tagged SDHC (Fig. 2C, anti-DDK panel, lanes 3 and 6). Thus, both SDHB WT and SDHB-loss imCCs successfully processed and expressed both control SDHC and the potentially rescuing SDHB subunits after transposition. In addition to successful gene transfer, transposed SDHB protein properly localized to mitochondria in SDHB-loss cells, colocalizing with the mitochondrial TOMM20 marker (Fig. 3A). We note that SDHB-rescued cells expressed excess SDHB, which became localized in the cytoplasm, and is therefore responsible for the red-tail phenotype observed in confocal microscopy. In our experience, this is not an uncommon phenomenon in gene manipulation assays involving protein overexpression. Localization of over-expressed rescuing SDHB protein (anti-DDK antibody) and the TOMM20 marker are shown in Fig. S2. We have no data on the cellular response to this unassembled subunit. Western blotting does not suggest ubiquitylation (Fig. 2C).

**Fig. 4.**
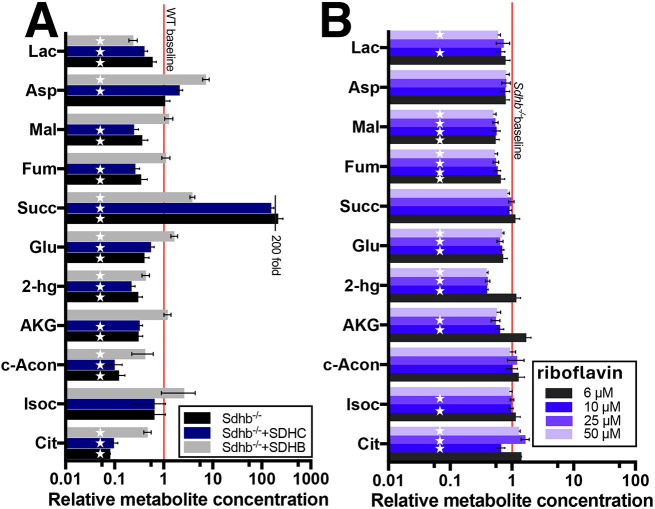
**Metabolite effects of gene restoration vs vitamin supplementation.** (A) *Sdhb* gene replacement substantially suppresses succinate accumulation in SDHB-loss imCC cells. Stars indicate 95% confidence interval for the ratio of the indicated metabolite in the indicated cell versus *Sdhb*^+/+^ cells being statistically different from 1.0. (B) Indicated levels of riboflavin supplementation of *Sdhb*^+/+^ or *Sdhb*^−/−^ cells does not suppress succinate accumulation in SDHB-loss imCC. Stars indicate 95% confidence interval for ratio of the indicated metabolite in the indicated cell versus untreated *Sdhb*^−/−^ cells being statistically different from 1.0. Ratio data are plotted on a log scale as mean±propagated error of the ratio, based on triplicate determinations normalized to protein content. Numerical data are provided in Tables S1 and S2.

Remarkably, various phenotypes of SDHB-loss imCCs were substantially or completely rescued by *Sdhb* gene replacement, but not by *Sdhc* gene replacement, as expected. Mitochondrial ultrastructure, as judged by transmission electron microscopy, shows restoration of a of more uniform mitochondrial electron density with a normal phenotype with absence of swollen mitochondria and pathological electron-dense mitochondrial deposits (Fig. 3B). This striking recovery is also reflected in the reestablishment of metabolic performance of the *Sdhb^−/−^*+SDHB imCC line compared to that seen for parent *Sdhb^−/−^*cells when subjected to Seahorse mitostress analysis (Fig. 3C; Fig. S1). Cell cycle analysis showed evidence of some recovery of normal cell cycle phases, although this not fully comparable to that in WT cells (Fig. 3D,E). Interestingly, the long overall cell doubling time of SDH-loss cells, perhaps reflecting both metabolic derangement and the burden of excess chromosomes in these aneuploid cells (Al Khazal et al., 2024), was not significantly shortened by *Sdhb* gene replacement (Fig. 3F). Quite remarkably, *Sdhb*, but not *Sdhc*, gene replacement, led to substantial normalization of intracellular metabolite levels (Fig. 4A; Table S1). In particular, succinate accumulation was reduced from over 200-fold to 4-fold relative to control cells. This result implies that all factors required for SDH complex assembly are available when the missing B subunit is provided (Maio et al., 2016; Rouault and Tong, 2008). Whether the reduction in accumulated succinate is sufficient to normalize dioxygenase functions will require further investigation. Thus, *Sdhb* gene replacement in SDHB-loss imCCs substantially reduces succinate accumulation, which is believed to be a primary oncogenic driver in PPGL.

### Attempts to rescue SDH activity in cultured SDHB-loss imCCs by riboflavin supplementation

Interestingly, riboflavin supplementation has previously been proposed to enhance residual SDHA flavinylation and SDH enzymatic function in disorders of oxidative phosphorylation, possibly reducing succinate levels by supporting conversion of accumulated succinate into fumarate by the catalytic SDHA subunit even in the absence of SDHB (Maio et al., 2016; Rouault and Tong, 2008). Previous studies have reported that 6 µM riboflavin was helpful *in vitro*. We tested this notion by culturing WT and SDHB-loss imCCs with 6, 10, 25 or 50 µM riboflavin for 2 weeks. Metabolomic analysis (Fig. 4B; Table S2) did not show decreased succinate accumulation under any condition, suggesting that enzyme activity of any remaining SDHA subunit could not be stimulated by increased flavinylation. Levels of fumarate, 2-ketoglutarate, 2-hydroxyglutarate and malate were reduced ∼2-fold by riboflavin treatment, but, as for succinate, levels of cis-aconitate, citrate and isocitrate were not reduced. Lactate levels were slightly depressed, consistent with the reported use of riboflavin to treat lactic acidosis (Dalton and Rahimi, 2001).

### Conclusions

Succinate accumulation is believed to be the primary oncogenic driver of SDH-deficient PPGL because of the ability of succinate to competitively inhibit dozens of cellular dioxygenases (Losman et al., 2020). This makes suppression of succinate accumulation a potential therapeutic or preventative strategy in PPGL. Here, we tested two conceptually simple tactics to suppress succinate accumulation in SDHB-loss imCCs with highly defective mitochondria: rescue of SDHB function by gene a replacement, or rescue of SDHA activity by stimulating SDHA flavinylation. We show that despite the fact that the mitochondrial biology of SDHB-loss cells is profoundly disrupted, normal processing and restoration of rescuing SDHB protein is readily achieved after gene replacement. This striking result demonstrates that assembly factors and other components required for electron transport chain protein organization remain available in spite of the severity of mitochondrial dysfunction in SDHB-loss cells. Substantial suppression of succinate accumulation by *Sdhb* gene replacement is an intriguing result, although therapeutic application of efficient *in vivo Sdhb* gene replacement is obviously difficult to envision with present technology in SDHB-deficient tumors. Whether the extent of succinate normalization upon SDHB replacement (∼200-fold to 4-fold) is sufficient to reverse tumorigenic signaling remains unknown.

It is uncertain what factors limit the ability of excess riboflavin supplementation to stimulate the catalytic activity of unassembled SDHA subunits in SDHB-loss imCCs in our experiments, although this capability has been suggested in other settings (Maio et al., 2016). This failure is regrettable, because high-dose vitamin therapy is known to be well-tolerated as a migraine treatment (Namazi et al., 2015). The inability of riboflavin to suppress succinate accumulation in cell culture should not rule out the possible clinical testing of this simple strategy in patients suffering from SDHx-deficient PPGL not involving SDHA.

Besides the strategies studied here, it is possible to screen for small molecules that suppress succinate accumulation by other mechanisms (Beimers et al., 2022; Braun et al., 2019). It remains to be determined whether relieving succinate accumulation would be a PPGL-preventative strategy in carriers of SDH-loss variants, or therapeutic in SDH-loss PPGL tumors. It is possible that advanced and metastatic PPGL cells accumulate additional mutations that allow them to escape succinate addiction by other mechanisms.

## MATERIALS AND METHODS

### Cell lines

WT and SDHB-loss immortalized mouse chromaffin imCCs (Letouze et al., 2013) were the maintained at 37°C, 95% humidity in room air (21% O_2_) with 5% CO_2_. Culture medium consisted of high-glucose DMEM with GlutaMAX™ (Gibco #10566016), 10% heat-inactivated FBS (Gibco #10082147) and a 0.5 mg/ml final concentration of penicillin-streptomycin antibiotics (Gibco #15140122). Additional supplements included 1 mM sodium pyruvate (Gibco #11140035), 10 mM HEPES buffer (Gibco #15630130) and nonessential amino acids [100 μM final concentration each of glycine, alanine, asparagine, aspartic acid, glutamic acid, proline and serine (Gibco #11140035)]. Cells were supplied with fresh medium every other day and replated based on their doubling rate when 80–90% confluency was reached. All experiments were performed within 14 passages from the initial seeding of frozen stocks. Morphology, growth characteristics, metabolism and transcription profiles of WT and SDH-loss imCCs have been recently characterized (Al Khazal et al., 2024).

### DNA transfer by transposition

We found SDHB-loss imCCs to be resistant to standard lentiviral gene transduction. We therefore resorted to Tol2 transposon technology (Clark et al., 2011) to move experimental transgenes into the cells of interest with co-transfection of the appropriate transposase. Mouse *Sdhb* and *Sdhc* were PCR amplified from OriGene plasmids MR203816 and MR201415, respectively. Gibson (NEB) cloning was used to insert *Sdhb* or *Sdhc* into the pKTol2C–EGFP plasmid (Clark et al., 2007; Addgene plasmid #85598; RRID:Addgene_8559). Both SDH subunit plasmids contain a MYC-DDK epitope tag on their 3′ termini to allow for western blot monitoring. TransIT-LT1 (Mirus) was used to co-transfect SDH or GFP, transposase and puromycin selection plasmids. Upon confirming transfection efficiency in SDHB-deficient imCCs through GFP expression, we replaced GFP with either SDHB or SDHC cDNA as the gene of interest.

### PCR genotyping assay

PCR was used to confirm transposition of WT rescue cDNA constructs encoding native N-terminal mitochondrial targeting peptides and C-terminal MYC-DDK epitope tags. PCR primers were as follows: SDHB primers 6973 (5′-CAT_4_G_2_CA_3_GA_2_T_2_C_2_TCGAGC_2_TGA_2_T_2_CTAC_2_ATG_2_CG_2_CG-ACG_2_TC-3′) and 6974 (5′-CATA_2_T_5_G_2_CAGAG_3_A_5_GATCT_3_A_3_C_2_T_2_ATCGTCGTCATC-3′) yielding a 945-bp PCR product, and SDHC primers 6972 (5′-CAT_4_G_2_CA_3_GA_2_T_2_C_2_TCGAGC_2_TG-A_2_T_2_CTAC_2_ATG_2_CTGCGCTCT_2_G-3′) and 6974 (5′-CATA_2_T_5_G_2_CAGAG_3_A_5_GATCT_3_A_3_C2T_2_ATCGTCGTCATC-3′), yielding a 606-bp PCR product.

The PCR protocol involved an initial denaturation at 98°C for 3 min, followed by 5 cycles at 94°C for 30 s, 50°C for 30 s and 72°C for 60 s, then 25 cycles at 94°C for 30 s, 65°C for 30 s and 72°C for 60 s, with a final elongation at 72°C for 5 min, and holding at 4°C.

### Western blotting

Assessment of endogenous and transposed SDHx protein expression was performed by standard western blotting. Cell pellets (3×10^6^ cells each) were lysed in 150 μl cold RIPA buffer containing protease and phosphatase inhibitor cocktail (Santa Cruz Biotechnology #sc-24948). Sample lysates were then incubated on ice for 30 min with gentle vortex mixing every 10 min. Following centrifugation at 15,000 ***g*** for 15 min, protein quantification was carried out using a BCA protein assay kit (Pierce #A55864). Samples were heated to 70°C for 10 min after the addition of reducing agent and an appropriate volume of 4× LDS denaturation buffer. Denatured samples (35 µg total protein) were subjected to electrophoresis through NuPAGE 10% Bis-Tris protein gels in MES-SDS running buffer at 150 V for 1 h. PVDF membrane transfer was performed according to the manufacturer's protocol for Novex Western transfer apparatus in NuPage transfer buffer containing 20% methanol at 4°C (30 V, 245 mA) for 90 min. Membranes were then blocked with 3% non-fat milk for 1 h at room temperature, followed by washing in Tris-buffered saline with 0.1% Tween 20 detergent (TBST) buffer. All blots were run simultaneously using the same protein extracts master mixes. A dilution buffer was prepared using 7.5 ml TBST, 2.5 ml 4% BSA and 250 μl 0.5% sodium azide was used with antibodies against SDHB (Abcam #ab175225, 1:5000), SDHC (Abcam #ab155999, 1:10,000) and DDK (Origene #TA592569, 1:1000) antibodies to detect their protein targets at predicted molecular masses [WT SDHB unprocessed (32 kDa) and processed (29 kDa); epitope-tagged SDHB: unprocessed (35 kDa) and processed (32kDa); WT SDHC unprocessed (18 kDa) and processed (15 kDa); epitope-tagged SDHC unprocessed 22 kD)a and processed (19 kDa)]. After 24 h of incubation at 4°C, blots were washed three times in TBST before staining with IRDye^®^ 680rd goat anti-rabbit IgG (#926-68071, 1:15,000) or 800 cw goat anti-mouse IgG secondary antibody (#926-32210, 1:15,000) antibodies in TBST with 3% non-fat milk for 1 h at room temperature prior to imaging. Total protein loading was confirmed by staining of replicate blots with Imperial protein stain (Thermo Fisher Scientific #24615) for 3–5 min followed by destaining overnight. All blots and protein loading gels were imaged with their appropriate settings on Amersham Typhoon 5 Biomolecular Imager. Original unmarked blot images are provided in Fig. S3.

### Mitochondrial electron microscopy

Sample preparation for electron microscopy was conducted at the Mayo Clinic Microscopy and Cell Analysis Core. 10^6^ cells per cell line were washed three times with PBS and fixed in 5 ml of McDowell Trump's fixative (Electron Microscopy Sciences #18030-05) using slow pipette mixing. Micrographs were captured using a JEOL 1400 Plus transmission electron microscope (JEOL, Inc., Peabody, MA) operating at 80 kV, equipped with a Gatan Orius camera (Gatan, Inc., Warrendale, PA).

### Cell cycle analysis

Cell cycle analysis was performed as previously published described with a few modifications (Al Khazal et al., 2024). In brief, three replicates of each line were plated 2 days prior to sample collection and harvested at 80% confluency, with ∼2×10^6^ cells per sample, by aspirating culture medium and washing three times with PBS. Cells were then fixed and permeabilized in ice-cold 70% ethanol for 3 days at −20°C. On day four, samples were washed three times in PBS prior to counting and resuspension of 10^6^ cells per sample in PBS. Two drops of Propidium Iodide Ready Flow™ Reagent (Invitrogen #R37169) were added to each sample, with unstained samples as controls. Samples were then incubated in the dark for 45 min before using a BD FACSymphony A3 Flow Cytometer (Becton, Dickinson and Co., Vernon Hills, IL, USA) to analyze 20,000 cells per sample.

### Doubling time analysis

The protocol for measuring average doubling time has been previously described (Al Khazal et al., 2024).

### Riboflavin treatment

Cells were supplemented with 6, 10, 25 or 50 µM riboflavin for 14 days. In brief, 6–50 µM final concentrations were achieved by diluting a 1 mM master concentration solution [9.41 mg riboflavin powder (Sigma-Aldrich #83-88-5) in 25 ml medium with no FBS added] and adding appropriate amounts. Cells were supplied with fresh medium daily, and were split according to their growth rate for each line when 80% confluence was reached.

### Seahorse XF cell mitochondrial stress test

Mitochondrial stress testing was performed in 20% O_2_ as previously described (Al Khazal et al., 2024).

### Metabolite quantification

Cells were grown to ∼80–90% confluency (∼1×10^6^ –3×10^6^ cells per 100-cm^2^ dish). Cells were washed three times with PBS to remove medium. Cells were frozen on dry ice and 1.5 ml chilled methanol (−20°C) was added to each dish. Cells were then scraped into methanol using a sterile plastic scraper with a rubber head. The resulting slurries were transferred to 2-ml conical tubes and frozen on dry ice. Samples were stored at −80°C prior to analysis. TCA cycle-related analytes were measured by gas chromatography/mass spectrometry in the Mayo Clinic Metabolomics Core Facility.

## Supplementary Material



10.1242/joces.263925_sup1 Supplementary information
