# Supplementary material for: Approaches to reduce succinate accumulation by restoration of succinate dehydrogenase activity in cultured adrenal cells
Source: J Cell Sci. 2025 May 12;138(9):jcs263925. doi: 10.1242/jcs.263925 (PMC12136175; doi:10.1242/jcs.263925)
Supplement: Supplementary information [file joces-138-263925-s1.pdf]

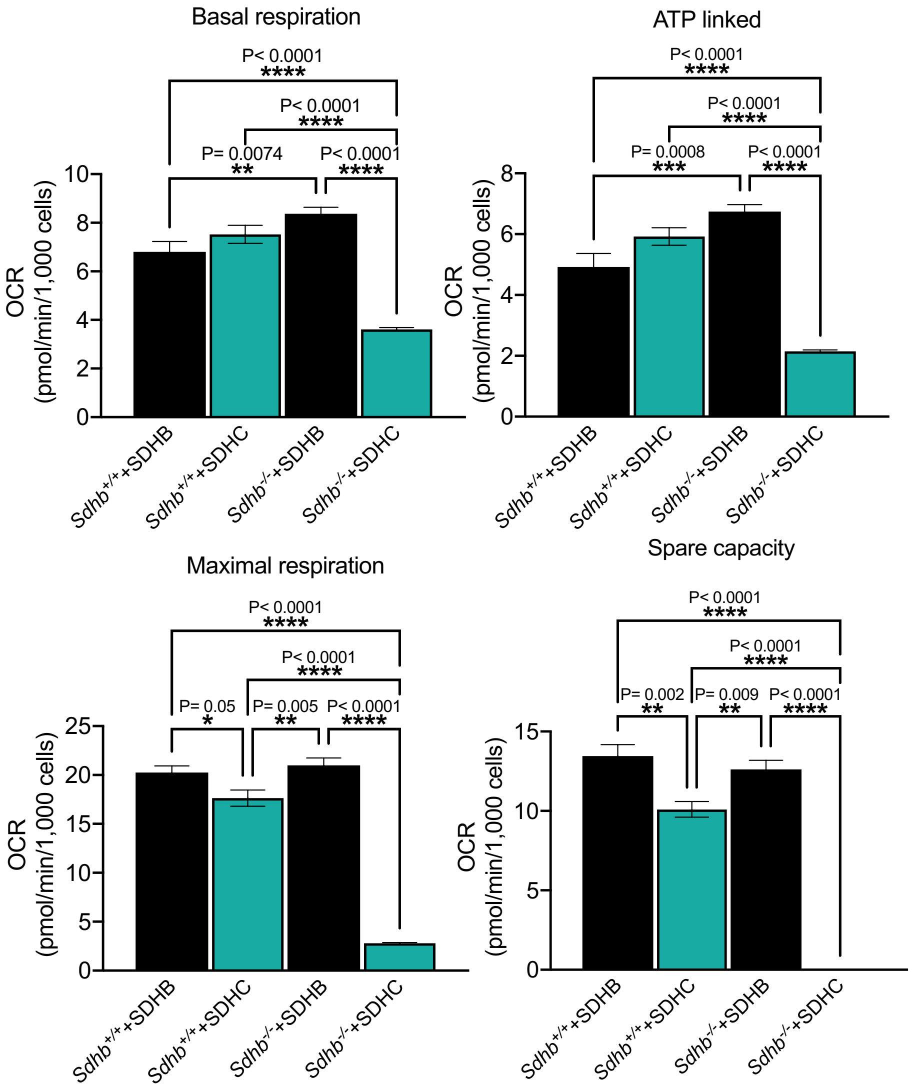

Fig. S1. Statistical breakdown of seahorse mitochondrial stress test analysis shown in Fig. 3C. Seahorse tests were performed in three replicates (n=7-8). Error bars represent mean ± SEM. P-values were calculated using multiple t-tests with Bonferroni correction. The number of asterisks indicates degree of significance.

Journal of Cell Science • Supplementary information

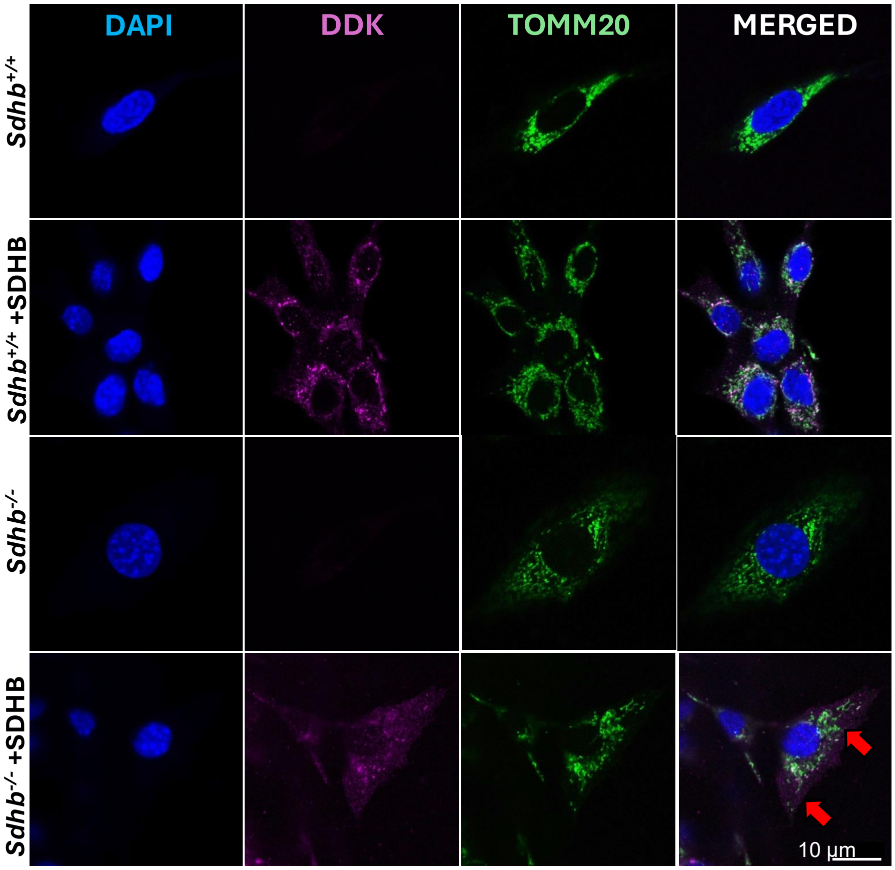

Fig. S2. Mitochondrial localization of DDK-tagged SDHB protein (magenta) based on co-localization with TOMM20 (green) as mitochondrial marker. Red arrows compare location of extra-mitochondrial SDHB in rescued *Sdhb*$^{-/-}$ compared to WT imCCs.

Journal of Cell Science • Supplementary information

Fig 2B

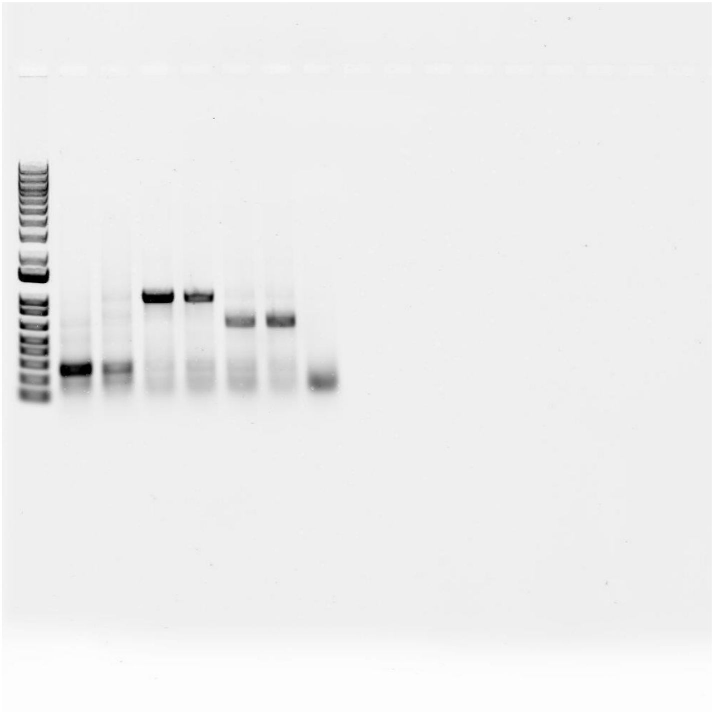

Fig 2C-1

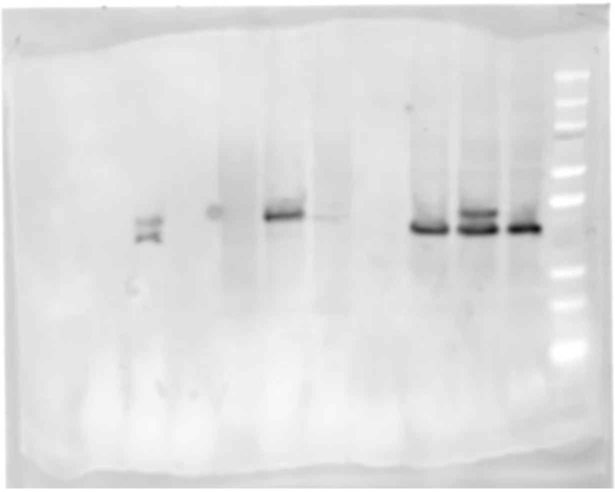

Fig 2C-2

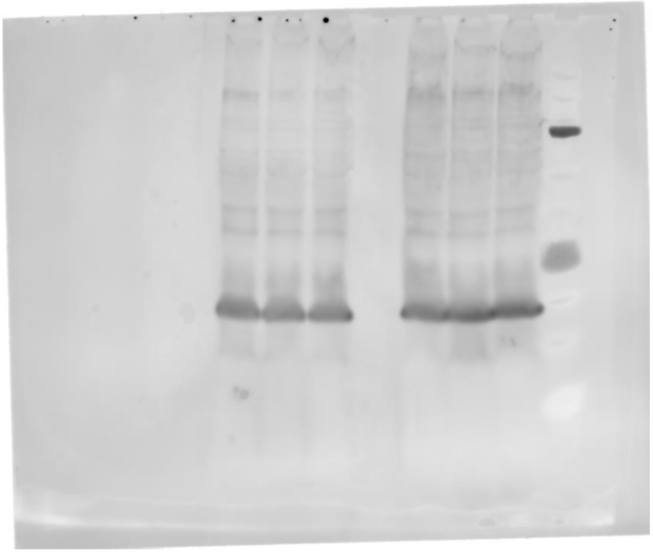

Fig 2C-3

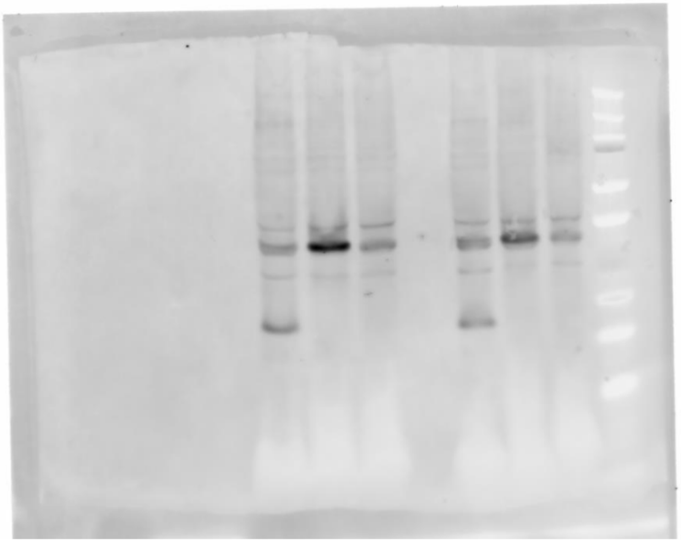

Fig 2C-4

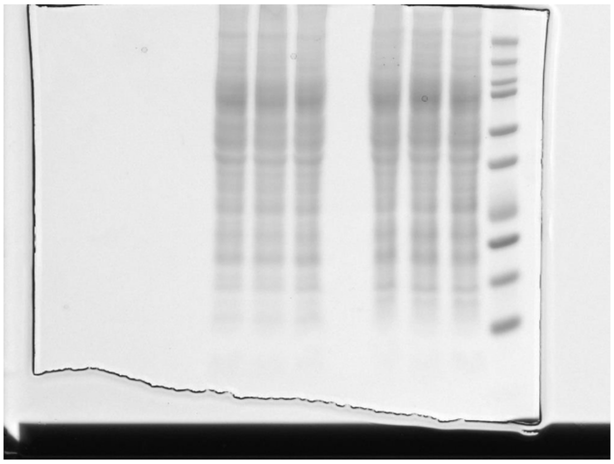

Fig. S3. Blot transparency data (unmarked original blot digital scans)

Table S1. TCA cycle-related metabolites in SDH-loss cells transposed with the indicated rescuing transgenes

Confidence Interval (CI) using t statistics (* indicates different from 1.0 at 95% confidence interval)

	Sdhb$^{-/-}$			Sdhb$^{-/-}$+SDHC			Sdhb$^{-/-}$+SDHB		
	value	error	95% CI	value	error	95% CI	value	error	95% CI
Cit	0.08	0.02	0.03, 0.13 *	0.10	0.02	0.05, 0.15 *	0.48	0.08	0.28, 0.68 *
Isoc	0.65	0.43	-0.4182, 1.7182	0.65	0.43	-0.42, 1.71	2.64	1.74	-1.68, 6.96
c-Acon	0.12	0.04	0.02, 0.22 *	0.10	0.04	0, 0.20 *	0.42	0.19	-0.05, 0.89 *
AKG	0.31	0.06	0.16, 0.46 *	0.33	0.04	0.23, 0.43 *	1.21	0.20	0.71, 1.71
2-hg	0.30	0.06	0.15, 0.45 *	0.22	0.04	0.12, 0.32 *	0.44	0.08	0.24, 0.64 *
Glu	0.41	0.09	0.19, 0.63 *	0.55	0.09	0.33, 0.77 *	1.66	0.26	1.01, 2.31 *
Succ	216.18	53.23	83.95, 348.41 *	156.67	19.64	107.88, 205.46 *	3.88	0.44	2.79, 4.97 *
Fum	0.35	0.11	0.08, 0.62 *	0.26	0.06	0.11, 0.41 *	1.12	0.21	0.60, 1.64
Mal	0.37	0.11	0.10, 0.64 *	0.25	0.06	0.11, 0.41 *	1.29	0.24	0.69, 1.89
Asp	1.07	0.27	0.40, 1.74	2.12	0.31	1.35, 2.89 *	7.33	1.11	4.57, 10.09 *
Lac	0.60	0.11	0.33, 0.87*	0.41	0.07	0.24, 0.58 *	0.24	0.05	0.12, 0.36 *

nmol metabolite per mg total protein (± standard deviation based on triplicate measurements) normalized to data for *Sdhb*$^{+/+}$ cells, with error propagation of the ratio. Lac: lactate; Su: succinate; Fum: fumarate; 2-KG: 2-ketoglutarate; Mal: malate; Asp: aspartate; 2-HG: 2-hydroxyglutarate; Glu: glutamate; c-Acon: cis-aconitate; Cit: citrate; iso-Cit: iso-citrate.)

Table S2. TCA cycle-related metabolites in SDH-loss cells treated with riboflavin

Confidence Interval (CI) using t statistics (* indicates different from 1.0 at 95% confidence interval)

riboflavin:	6 μM			10 μM			25 μM			50 μM		
	value	error	95% CI	value	error	95% CI	value	error	95% CI	value	error	95% CI
Cit	1.43	0.23	0.86, 2.00	0.67	0.10	0.42, 0.92 *	1.66	0.25	1.04, 2.28 *	1.31	0.05	1.19, 1.43 *
Isoc	1.16	0.20	0.66, 1.66	0.97	0.08	0.77, 1.17	1.00	0.07	0.83, 1.17 *	0.92	0.08	0.72, 1.13
c-Acon	1.29	0.30	0.79, 1.79	1.01	0.22	0.46, 1.56	1.21	0.36	0.32, 2.10	0.94	0.21	0.42, 1.46
AKG	1.70	0.33	0.88, 2.52	0.65	0.09	0.43, 0.87 *	0.55	0.09	0.33, 0.77 *	0.58	0.08	0.38, 0.78
2-hg	1.18	0.21	0.66, 1.70	0.40	0.02	0.35, 0.45 *	0.41	0.03	0.34, 0.48 *	0.39	0.02	0.34, 0.44 *
Glu	0.72	0.12	0.42, 1.02	0.70	0.05	0.58, 0.82 *	0.65	0.07	0.48, 0.82 *	0.70	0.04	0.60, 0.80 *
Succ	1.14	0.19	0.67, 1.61	0.92	0.07	0.75, 1.09	0.98	0.10	0.73, 1.23	0.86	0.06	0.71, 1.01
Fum	0.66	0.10	0.41, 0.91 *	0.59	0.07	0.42, 0.76 *	0.55	0.06	0.40, 0.70 *	0.53	0.06	0.38, 0.68 *
Mal	0.55	0.07	0.38, 0.72 *	0.57	0.08	0.37, 0.77 *	0.54	0.05	0.42, 0.66 *	0.50	0.05	0.38, 0.62 *
Asp	0.80	0.11	0.53, 1.07	0.79	0.13	0.47, 1.11	0.81	0.13	0.49, 1.13	0.81	0.10	0.56, 1.06
Lac	0.79	0.14	0.44, 1.14	0.67	0.10	0.42, 0.92 *	0.74	0.18	0.49, 1.13	0.60	0.06	0.45, 0.75 *

nmol metabolite per mg total protein (± standard deviation based on triplicate measurements) normalized to data for untreated *Sdhb*$^{-/-}$ cells, with error propagation of the ratio. Lac: lactate; Su: succinate; Fum: fumarate; 2-KG: 2-ketoglutarate; Mal: malate; Asp: aspartate; 2-HG: 2-hydroxyglutarate; Glu: glutamate; c-Acon: cis-aconitate; Cit: citrate; iso-Cit: iso-citrate.)
