# Supplementary material for: Approaches to reduce succinate accumulation by restoration of succinate dehydrogenase activity in cultured adrenal cells
Source: J Cell Sci. 2025 May 12;138(9):jcs263925. doi: 10.1242/jcs.263925 (PMC12136175; doi:10.1242/jcs.263925)
Supplement: Supplementary file 1 [file joces-138-263925_review_history.pdf]

Journal of Cell Science

Approaches to reduce succinate accumulation by restoration of succinate dehydrogenase activity in cultured adrenal cells

Fatimah Al Khazal, Leili Rahimi, Fan Feng, Nicole Becker, Clifford Folmes, Judith Favier and Louis J. Maher
DOI: 10.1242/jcs.263925

Editor: Daniel Billadeau

Review timeline

Original submission:	12 February 2025
Editorial decision:	18 March 2025
First revision received:	28 March 2025
Accepted:	3 April 2025

Original submission

First decision letter

MS ID#: jcs.263925

MS TITLE: Approaches to reduce pathological succinate accumulation by restoration of succinate dehydrogenase activity in cultured adrenal cells

AUTHORS: Fatimah Al Khazal; Leili Rahimi; Fan Feng; Nicole Becker; Clifford Folmes; Judith Favier; Louis J Maher

We have now reached a decision on the above manuscript.

To see the reviewers' reports and a copy of this decision letter, please go to:

As you will see, the reviewers gave favourable reports but raised some critical points that will require amendments to your manuscript. I hope that you will be able to carry these out because I would like to be able to accept your paper, depending on further comments from reviewers.

Reviewer 1

Advance summary and potential significance to field

In the manuscript Khazal et al investigate strategies to rescue the mitochondrial and metabolic phenotypes of a mouse adrenal medulla derived cell line (imCCs) where the pheochromocytoma / paraganglioma linked protein succinate dehydrogenase subunit B (SDHB) is lost. Firstly, they heterologously express SDHB but via transposon technology. In addition to this genetic approach the authors treat cells with the small molecule drug riboflavin. They demonstrate that SDHB replacement can rescue aspects of mitochondrial dysfunction and reduce succinate accumulation. Contrastingly, riboflavin supplementation does not rescue.

Comments for the author

The paper is clearly written with convincing and well-presented data. Nevertheless I have a number of comments the authors should address.

© 2025. Published by The Company of Biologists under the terms of the Creative Commons Attribution License (https://creativecommons.org/licenses/by/4.0/).

1. As a negative control for rescue of the SDHB-loss imCCs, by Sdhb gene replacement, cells were also transposed with DNA encoding Sdhc. While this is a reasonable control to include, I feel a better approach would have been to introduce a loss of function missense mutation into the sdhb cDNA. A number of clinically occurring missense mutations in SDHB have been identified, while some of these mutations impact protein half-life, this control would have further strengthened the work.

2. Figure 3A shows magnification of cell images. This is not useful relative to including a scale bar. The authors should replace magnification with scale bars.
Figure 3A would also benefit from inclusion of some zoom panels to allow the reader to better see the colocalization of Tom20 and SDHB. These should be included.
Furthermore, there does seem to be variability between the degree of Tom20 colocalization with SDHB in the cells with different SDHB expression. Does the heterologous expressed SDHB colocalize to a greater or lesser degree with Tom20?
The authors should perform the immunofluorescent labelling with anti-myc as well and look at colocalization with SDHB and Tom20.
Finally, the extra mitochondrial SDHB (shown with red arrows), does this colocalize with any other cell structures or proteins? For example, is it targeted for degradation by the UPS or autophagy-lysosomal pathway.

3. Also, in Figure 3A why are the Sdhb plus exogenous B cells so much smaller. Is this real or is this an image selection issue? The authors should quantify these apparent differences in sizes of the cells with different levels of SDHB expression and if this is significant, suggest possible explanations.

4. From Figure 3B the authors report 'Mitochondrial ultrastructure judged by transmission electron microscopy shows restoration of a of more uniform mitochondrial electron density with a normal phenotype with absence of swollen mitochondria and pathological electron-dense mitochondrial deposits, and restoration'. An attempt should be made to quantify these changes, such that the degree of rescue can be better judged.

5. Figure 4 indicates metabolite levels in WT imCCs cells, by a red line (labelled baseline). Numeric data for this should be included in the supplemental table. Why is statistical significance not indicated in Figure 4. This should be included, the authors could just give p values relative to Sdhb-/-, for example.

<u>Reviewer 2</u>

Advance summary and potential significance to field

The accumulation of succinate in succinate dehydrogenase (SDH)-deficient pheochromocytomas and paragangliomas (PCPG) is thought to contribute to the development and pathogenicity of these tumors. However, the precise effects of succinate and their underlying mechanisms are incompletely understood. Several SDH-deficient mammalian cell lines have been developed to study the pathobiology of SDH-deficient PCPG but there have been few, if any, successful attempts to restore SDH activity in these models for basic research or as a potential therapy. This paper tested two approaches to accomplish that goal in Sdhb-loss immortalized mouse chromaffin cells: reintroduction of Sdhb using the Tol2 transposon system, and riboflavin supplementation aimed to stimulate enzymatic activity of residual Sdha by increasing flavination. The former approach worked in several respects (starting with the technical achievement of properly localizing the transposed protein to the mitochondria) and the latter did not.
This paper is an important contribution to PCPG research in providing a new tool and path to pursue.

Comments for the author

This paper is an important contribution to PCPG research in providing a new tool and path to pursue. I offer mostly minor comments.

© 2025. Published by The Company of Biologists under the terms of the Creative Commons Attribution License (https://creativecommons.org/licenses/by/4.0/).

1. Some of the figures and tables do not include unmodified Sdhb-/- or unmodified Sdhb+/+ cells, so the reader cannot be entirely sure of the extent to which the +/+ phenotype is restored. The Sdhb-/- +SDHC condition is a good control but it would be informative to compare the unmodified cells as well.
2. With particular regard to the above, in Fig 3B the Sdhb++ +SDHC cells (column 2) appear less healthy than Sdhb+/+ +SDHB cells (column 1), so using Sdhb-/- +SDHC as a control for Sdhb-/- +SDHB could exaggerate some effects of restored SDHB. How is the cytoplasmic vacuolization in columns 2 and 3 (ER swelling?) explained?
3. Were cultures used for cell cycle analysis at the same plating density and same number of days post-trypsinization?
4. The riboflavin studies are essentially presented as negative data but are nonetheless of interest and raise a few additional questions that could be addressed: What is the status of SDHAF2 in the Sdhb-/- cells? What is the status of COX? (SDHAF2 affects assembly of both SDH and COX, the latter independently of SDH (DOI https://doi.org/10.1016/j.mito.2024.101952). Is there an additive or synergistic effect of riboflavin supplementation with Sdhb restoration?

First revision

Author response to reviewers' comments

Reviewer 1: SUMMARY OF THE ADVANCE MADE IN THIS PAPER AND ITS POTENTIAL SIGNIFICANCE TO THE FIELD

In the manuscript Al Khazal et al. investigate strategies to rescue the mitochondrial and metabolic phenotypes of a mouse adrenal medulla derived cell line (imCCs) where the pheochromocytoma / paraganglioma linked protein succinate dehydrogenase subunit B (SDHB) is lost. Firstly, they heterologously express SDHB but via transposon technology. In addition to this genetic approach the authors treat cells with the small molecule drug riboflavin. They demonstrate that SDHB replacement can rescue aspects of mitochondrial dysfunction and reduce succinate accumulation. Contrastingly, riboflavin supplementation does not rescue.

We appreciate this supportive and accurate description of our report.

SUGGESTIONS TO AUTHORS

The paper is clearly written with convincing and well-presented data. Nevertheless I have a number of comments the authors should address.

We thank the referee for their compliment and suggestions.

1. As a negative control for rescue of the SDHB-loss imCCs, by Sdhb gene replacement, cells were also transposed with DNA encoding Sdhc. While this is a reasonable control to include, I feel a better approach would have been to introduce a loss of function missense mutation into the sdhb cDNA. A number of clinically occurring missense mutations in SDHB have been identified, while some of these mutations impact protein half-life, this control would have further strengthened the work.

Response: We would like to thank the reviewer for the thoughtful suggestion of introducing a loss-of-function missense mutation into the Sdhb cDNA as additional negative controls. In fact, we considered such an approach initially, and rejected it for practical reasons. Our chosen approach interrogates two extremes: complete loss of WT SDHB expression and complete restoration of WT SDHB expression. These conditions represent the clearest binary comparison. As such, SDHC transduction as negative control serves all of our desired goals related to controlling for effects of transfection, transposition, drug selection, etc. Our practical concern that led us to avoid introducing e.g. SDHB missense variants involved, among other concerns, the need to create the variant constructs and then independently document their lack of function in our cell culture system. These efforts would have involved identification,

© 2025. Published by The Company of Biologists under the terms of the Creative Commons Attribution License (https://creativecommons.org/licenses/by/4.0/).

introduction, and characterization of a clinically relevant missense mutation, along with repeating parental line characterization. It was our conclusion that mock restoration with SDHC provides a cleaner experiment whose results are easier to interpret. We therefore have respectfully declined to pursue the suggested additional approach. We agree that future studies could be undertaken to investigate any potential differences between loss-of-function SDHB missense mutations and complete gene deletion.

We now add to the introduction section of the manuscript this statement:

We opted for Sdhc replacement as an uncomplicated negative control, though future studies could compare this result to replacement with validated loss-of-function missense Sdhb constructs.

2. Figure 3A shows magnification of cell images. This is not useful relative to including a scale bar. The authors should replace magnification with scale bars.
Figure 3A would also benefit from inclusion of some zoom panels to allow the reader to better see the colocalization of Tom20 and SDHB. These should be included.

Response: We appreciate this referee suggestion. Magnification has been replaced with scale bars as suggested. We have found that 200x magnification, the maximum we can achieve with our available instrumentation, does not provide a clearer view of localization, but has the liability of limiting visualization to only a single cell at a time. Thus, we find that the presented magnification data optimizes visibility of the two key points we demonstrate: 1) restoration of SDHB and its colocalization with the mitochondria, and 2) the presence of excess extramitochondrial SDHB protein in rescued experimental lines, not as evident in WT cultures.

3. Variability is observed in the degree of Tom20 colocalization with SDHB in cells with different levels of SDHB expression. Does the heterologous expressed SDHB colocalize to a greater or lesser degree with Tom20?

Response: We appreciate the reviewer's attention in noting differences in Tom20 and SDHB colocalization between the parental cell lines and SDHB-rescued lines. Our assessment is that SDHB-rescued cells express excess SDHB, which becomes localized outside the mitochondria (in the cytoplasm), and is therefore responsible for the red-tail phenotype observed in confocal microscopy. In our experience, this is not an uncommon phenomenon in gene manipulation assays involving protein overexpression.

To clarify this point, we add to the results section the following wording:

We note that SDHB-rescued cells express excess SDHB, which becomes localized in the cytoplasm, and is therefore responsible for the red-tail phenotype observed in confocal microscopy. In our experience, this is not an uncommon phenomenon in gene manipulation assays involving protein overexpression.

4. The authors should perform the immunofluorescent labelling with anti-myc as well and look at colocalization with SDHB and Tom20.

Response: We appreciate this referee suggestion. We have thoroughly assessed the localization pattern of SDHB rescue lines using anti-SDHB anti-DDK antibodies, and antibodies against Tom20. However, we found that co-staining with all three reagents was not ideal challenging because the antibodies in which we have greatest confidence (anti-SDHB and anti-DDK) are both mouse antibodies with high cross-reactivity. We now provide anti-DDK staining data for parental lines and SDHB rescue lines as supplementary Fig. S2.

We have added the following wording to the results section:

Localization of over-expressed rescuing SDHB protein (anti-DDK antibody) and the TOMM220 marker are shown in Supplementary Fig. S2.

© 2025. Published by The Company of Biologists under the terms of the Creative Commons Attribution License (https://creativecommons.org/licenses/by/4.0/).

5. Finally, the extra mitochondrial SDHB (shown with red arrows), does this colocalize with any other cell structures or proteins? For example, is it targeted for degradation by the UPS or autophagy-lysosomal pathway?

Response: We thank the referee for this intriguing comment. Our impression is that the SDHB rescue lines express excess cytoplasmic SDHB due to gene overexpression or cellular stress, both of which are commonly reported in gene manipulation studies. We have no data on the question of any processing affecting this unassembled subunit.

We have added to the results the following sentence:

We have no data on the cellular response to this unassembled subunit. Western blotting does not suggest ubiquitylation.

6. Also, in Figure 3A why are the Sdhb plus exogenous B cells so much smaller. Is this real or is this an image selection issue? The authors should quantify these apparent differences in sizes of the cells with different levels of SDHB expression and if this is significant, suggest possible explanations.

Response: The referee's astute observation is correct. Our recently published detailed characterization of imCC lines (Al Khazal, Bhat et al.), which we cite as a preliminary source for this work, details size and plating behavior differences between WT and SDHB-loss immortalized adrenal cell lines. In fact, SDHB-loss cells are approximately three times larger than WT cells and each cell occupies more surface area when plated. The two imaged *Sdhb*$^{+/+}$ +SDHB cells are also exiting cell division, possibly contributing to mitochondrial localization near the nucleus.

7. From Figure 3B the authors report 'Mitochondrial ultrastructure judged by transmission electron microscopy shows restoration of a of more uniform mitochondrial electron density with a normal phenotype with absence of swollen mitochondria and pathological electron-dense mitochondrial deposits, and restoration'. An attempt should be made to quantify these changes, such that the degree of rescue can be better judged.

Response: The very valid suggestion of the referee is appreciated. Our intention with these TEM micrographs was to make a general assessment of mitochondrial health. Please note that these random section images cannot be used to estimate mitochondrial volume, mitochondrial count, or surface area/cristae area, as these can only be accurately assessed with 3D TEM. We can, however, make a general assessment of mitochondrial health compared to the selected controls and previously reported parental lines. A more complete comparison of mitochondrial volume density in WT and SDHB-loss imCC was document in our prior paper (Al Khazal, Bhat, et al.) For the purpose of answering the question, "Does lowering the burden of succinate accumulation affect mitochondrial health?" we believe the provided TEM images provide a sufficient answer.

5. Figure 4 indicates metabolite levels in WT imCCs cells, by a red line (labelled baseline). Numeric data for this should be included in the supplemental table. Why is statistical significance not indicated in Figure 4. This should be included, the authors could just give p values relative to Sdhb-/-, for example.

Response: We appreciate this helpful referee suggestion and we agree that providing insight into quantitative statistical significance is valuable. We have therefore modified Fig. 4 and Supplemental Tables S1 and S2 accordingly. The figures report metabolite data (mass normalized to protein) as an appropriate ratio between the metabolite in the experimental cells vs. the appropriate control cells, and the ratio results are plotted on a log scale with ratio error properly propagated from the standard deviation of the triplicate determinations in each case. Per the referee's suggestion, we now have calculated the 95% confidence interval for the ratio for each metabolite, indicating in both Fig. 4 (stars) and supplementary tables S1 and S2 (asterisks) those ratios that are statistically significantly different (higher or lower) from 1.0.

The legend for Fig 4 has correspondingly been reworded:

© 2025. Published by The Company of Biologists under the terms of the Creative Commons Attribution License (https://creativecommons.org/licenses/by/4.0/).

Figure 4. Metabolite effects of gene restoration vs. vitamin supplementation. A. Sdhb gene replacement substantially suppresses succinate accumulation in SDHB-loss imCC cells. Stars indicate 95% confidence interval for ratio of the indicated metabolite in the indicated cell vs. Sdhb$^{+/+}$ cells is statistically different from 1.0. B. Indicated levels of riboflavin supplementation of Sdhb $^{+/+}$ or Sdhb $^{-/-}$ cells does not suppress succinate accumulation in SDHB-loss imCC. Stars indicate 95% confidence interval for ratio of the indicated metabolite in the indicated cell vs. untreated Sdhb$^{-/-}$ cells is statistically different from 1.0. Ratio data are plotted on a log scale as mean ± propagated error of the ratio, based on triplicate determinations normalized to protein content. Numerical data are provided in Supplementary Tables S1 and S2.

For Supplementary Tables S1 and S2 we also now indicate:

Confidence Interval (CI) using t statistics (indicates different from 1.0 at 95% confidence interval).*

Reviewer 2: SUMMARY OF THE ADVANCE MADE IN THIS PAPER AND ITS POTENTIAL SIGNIFICANCE TO THE FIELD

The accumulation of succinate in succinate dehydrogenase (SDH)-deficient pheochromocytomas and paragangliomas (PCPG) is thought to contribute to the development and pathogenicity of these tumors. However, the precise effects of succinate and their underlying mechanisms are incompletely understood. Several SDH-deficient mammalian cell lines have been developed to study the pathobiology of SDH-deficient PCPG but there have been few, if any, successful attempts to restore SDH activity in these models for basic research or as a potential therapy. This paper tested two approaches to accomplish that goal in Sdhb-loss immortalized mouse chromaffin cells: reintroduction of Sdhb using the Tol2 transposon system, and riboflavin supplementation aimed to stimulate enzymatic activity of residual Sdha by increasing flavination. The former approach worked in several respects (starting with the technical achievement of properly localizing the transposed protein to the mitochondria) and the latter did not.

This paper is an important contribution to PCPG research in providing a new tool and path to pursue.

Response: We appreciate these very supportive reviewer comments.

SUGGESTIONS TO AUTHORS

This paper is an important contribution to PCPG research in providing a new tool and path to pursue. I offer mostly minor comments.

These reviewer comments are appreciated.

1. Some of the figures and tables do not include unmodified Sdhb-/- or unmodified Sdhb+/+ cells, so the reader cannot be entirely sure of the extent to which the +/+ phenotype is restored. The Sdhb-/- +SDHC condition is a good control but it would be informative to compare the unmodified cells as well.

Response: We appreciate this referee suggestion. Parental line characterization, including Seahorse assay, mitochondrial EM imaging, cell cycle analysis, and metabolite measurements, has recently been published and referenced as preliminary work for this project (Al Khazal, Bhat, et al https://doi.org/10.1186/s40170-024-00369-9). Here we limited our comparisons to aaspects not previously addressed in earlier work, such as SDHB confocal staining. This has now been clarified in the methods section where we have added the wording:

Morphology, growth characteristics, metabolism, and transcription profiles of WT and SDH-loss imCCs have been recently characterized (Al Khazal et al., 2024).

© 2025. Published by The Company of Biologists under the terms of the Creative Commons Attribution License (https://creativecommons.org/licenses/by/4.0/).

2. With particular regard to the above, in Fig 3B the Sdhb++ +SDHC cells (column 2) appear less healthy than Sdhb+/+ +SDHB cells (column 1), so using Sdhb-/- +SDHC as a control for Sdhb-/- +SDHB could exaggerate some effects of restored SDHB. How is the cytoplasmic vacuolization in columns 2 and 3 (ER swelling?) explained?

Response: We appreciate this referee comment. We note that the provided micrographs were selected to provide a general assessment of sub-cellular compartments, the TEM sample preparation process involves cell harvest, centrifugation, resin embedding and ultrathin (60-80 nm) sectioning of cell clusters. Thus, images are not captured on surface-grown monolayers and the thin visualized cell section that could fall anywhere across the cell body. Cytoplasmic volume is expected to be exaggerated or underestimated in 1D micrographs based on such preparations.

3. Were cultures used for cell cycle analysis at the same plating density and same number of days post-trypsinization?

Response: We appreciate the opportunity to clarify this important point. The referee is correct. Clarifying text has been added to the method section to reflect this:

In brief, three replicates of each line were plated 2 d prior to sample collection and harvested at 80% confluency, with approximately two million cells per sample, by aspirating culture medium and washing three times with PBS. Cells were then fixed and permeabilized in ice-cold 70% ethanol for three d at -20°C.

4. The riboflavin studies are essentially presented as negative data but are nonetheless of interest and raise a few additional questions that could be addressed: What is the status of SDHAF2 in the Sdhb-/- cells? What is the status of COX? (SDHAF2 affects assembly of both SDH and COX, the latter independently of SDH (DOI https://doi.org/10.1016/j.mito.2024.101952). Is there an additive or synergistic effect of riboflavin supplementation with Sdhb restoration?

Response: This valid referee inquiry is appreciated. Our most recently published data suggest that SDHB-loss cells have lower expression of COX, while our published RNA-seq data deposit indicates no significant change in SDHAF2 expression, with a ~0.3975 $\log_2$ (FC) and a p-value of 1.118×10^{-16}.
https://doi.org/10.1186/s40170-024-00369-9

We plan future work to address this question more thoroughly by investigating NADH and NAD+ and redox status in the context of SDH-loss.

Editor suggestions:

1. Use graphs that allow the reader to see the true data spread (i.e. box and whisker plots, super plots, etc. See PMID: 32346721 for more information.). If using box-and-whisker plots, please also state what the whiskers represent. See here for helpful information.

Response: Figure 3F has been converted to box and whisker format.

2. Use appropriate statistics with the sample size representing biological replicates rather than technical replicates

Response: no technical samples were used in this study.

3. Provide, if using western blots, one additional supplementary figure (as a single PDF or TIFF file). It will not contribute towards your supplementary figure limit. If you provided this at submission and named it 'Blot transparency', please now number this supplementary figure and combine it with the rest of the supplementary material pdf. Please ensure that you cite this figure in your Materials & Methods section, ideally in the western blots paragraph.

Response: Images files of original western blots are now included in a series of “blot transparency” pages in the supplemental materials.

© 2025. Published by The Company of Biologists under the terms of the Creative Commons Attribution License (https://creativecommons.org/licenses/by/4.0/).

**Please note that, as per our policy, HIGH-CONTRAST GELS AND BLOTS ARE UNACCEPTABLE (i.e. no white backgrounds). If you supply gels/blots with white backgrounds, we will ask you for the original data for verification, which will delay the processing of your manuscript.

Response: original files have been provided with no digital alterations

The length limit for Research Articles is 8000 words, and 3000 for Short Reports. This word count does not include the title page, abstract, materials & methods, or reference list but does include figure legends.

Response: length is within range.

Please collate supplementary figures and tables into a single PDF file.

Response: This condition has been met

Title limit

We have a strict limit for the title of the paper to be under 120 characters, including spaces. As your title length (132) currently exceeds this, we would appreciate it if you could reduce the length of your title before you re-upload your files.

Response: We appreciate this title length directive. We have edited our title to be: "Approaches to reduce succinate accumulation by restoration of succinate dehydrogenase activity in cultured adrenal cells" (120 characters and spaces)

Section Order

Article sections should appear in the following order: Introduction, Results, Discussion, Materials and methods.

Response: condition has been met

Results and discussion sections combined. We require these to be in two separate sections, so could you please separate these before re-uploading your files.

Response: condition has been met

Submission checklist

We are now requesting that authors fill in and upload a submission checklist with their manuscript. This form asks authors to confirm that they have followed best practice guidelines regarding experimental subjects, data reporting and statistics. The checklist is based on the NIH Principles and Guidelines for Reporting Preclinical Research and is intended to help ensure high standards for reporting and to aid reproducibility.

Please fill in and upload the submission checklist into the supplemental files area. You can find a copy of the checklist here.

Response: Complete and included.

Second decision letter

MS ID#: jcs.263925R1

© 2025. Published by The Company of Biologists under the terms of the Creative Commons Attribution License (https://creativecommons.org/licenses/by/4.0/).

MS TITLE: Approaches to reduce pathological succinate accumulation by restoration of succinate dehydrogenase activity in cultured adrenal cells

AUTHORS: Fatimah Al Khazal; Leili Rahimi; Fan Feng; Nicole Becker; Clifford Folmes; Judith Favier; Louis J Maher
ARTICLE TYPE: Short Report

I am happy to tell you that your manuscript has been accepted for publication in Journal of Cell Science, pending standard publication integrity checks.

© 2025. Published by The Company of Biologists under the terms of the Creative Commons Attribution License (https://creativecommons.org/licenses/by/4.0/).
